# New European records of *Ditomyiamacroptera* Winnertz (Diptera: Ditomyiidae) with notes on its distribution

**DOI:** 10.3897/BDJ.6.e24857

**Published:** 2018-04-24

**Authors:** Olavi Kurina, Peter Chandler

**Affiliations:** 1 Estonian University of Life Sciences, Institute of Agricultural and Environmental Sciences, Kreutzwaldi 5D, Tartu, Estonia Estonian University of Life Sciences, Institute of Agricultural and Environmental Sciences, Kreutzwaldi 5D Tartu Estonia; 2 606B Berryfield Lane, Melksham, Wilts SN12 6EL, United Kingdom 606B Berryfield Lane Melksham, Wilts SN12 6EL United Kingdom

**Keywords:** Diptera, Ditomyiidae, Europe, distribution, mycetophagy

## Abstract

**Background:**

*Ditomyiamacroptera* Winnertz, the rarest European ditomyiid fly, is known only by a few specimens across the collections. Besides a single male specimen from Sakhalin Island, all other documented records are from Central Europe.

**New information:**

New records of *Ditomyiamacroptera* Winnertz from Bulgaria and France are presented representing the second rearing event after its initial description. Illustrations of the general facies and male terminalia are given. The study of old collection material reveals exclusion of the species from the Belgian list and allows us to discuss the origin of two specimens in the collection of C.R. Osten-Sacken in St. Petersburg, Russia.

## Introduction

Ditomyiidae is a worldwide distributed family of Diptera, represented by nearly a hundred described species in 9 extant genera (cf. www.sciaroidea.info). Four species in two genera are known to occur in Europe. Both genera – *Ditomyia*Winnertz, 1846 and *Symmerus* Walker, 1848 – are represented by two species (Chandler 2004). *Ditomyiafasciata* (Meigen, 1818) is a widely distributed species in Europe, although not very common in samples and is also recorded from the Eastern Palaearctic (Zaitzev 1994, Chandler 2004). On the contrary, *Ditomyiamacroptera* (Winnertz, 1852) is a Palaearctic species, exceptionally rare in Europe, so far known only by a relatively small number of specimens. Having been described by Winnertz (Winnertz 1852: 55) from a single female specimen collected in Aachen (Germany), the species is subsequently recorded from Poland (Mikołaczyk 1991), Germany (Zaitzev 1978), Czech and Slovak Republics (Ševčík 2004, Ševčík et al. 2005, Ševčík et al. 2013), Italian mainland (Dahl et al. 1995, Kurina 2008) and Belgium (Gosseries 1991). One male specimen has been also recorded by Okada (Okada 1936) from the Eastern Palaearctic: Sakhalin Island (but see discussion below). According to the original description *D.macroptera* was reared from *Phellinusigniarius* (Winnertz 1852: 55) and there have not been additional data on the biology of this species up to the present. The European congener – *D.fasciata*– is known from different bracket fungi but most frequently from *Trametesversicolor* (Jakovlev 1994), *Bjerkanderaadusta* and *Polyporus* species (Ševčík 2010).

During recent years, new material has come into the authors’ possession that initiated the current communication. Here we present the new country records from Bulgaria and France, along with comments on earlier ones resulting in changes in the distribution range of the species.

## Materials and methods

**Figure 1.**
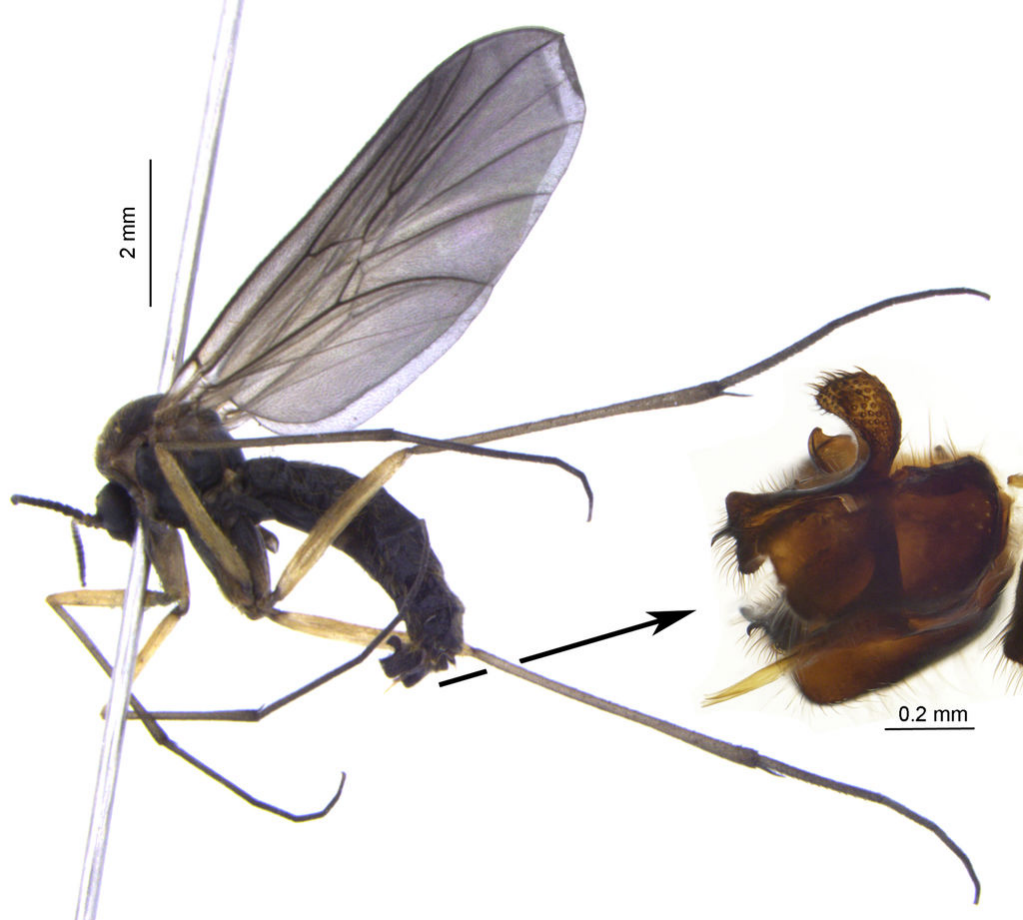
*Ditomyiamacroptera* Winnertz, male specimen from Bulgaria: general habitus in lateral view (left) and close lateral view of terminalia (right).

The Bulgarian material (Fig. 1) is reared from fruiting bodies of *Phellinusalni*, which were collected in an old orchard in village of Ilindentsi, South Western part of the country. The French material was collected in the southwest of the Jura Mountain Range in France, on the French-Swiss border with Malaise traps in 2011, but only received for examination by PC in 2015. Samples from 12 Malaise traps at this site, operated from 2009 to 2011, produced 112 species of fungus gnats, but only two specimens of *D.macroptera* were present. The two traps involved were situated about 100 m apart in a historically drained bog area at 850 m altitude, at a site where *Phellinusigniarius* has been recorded; both specimens lack legs and the male lacks antennae.

The material is deposited in following institutional and private collections:

IZBE – Institute of Agricultural and Environmental Sciences, Estonian University of Life Sciences [former Institute of Zoology and Botany], Tartu, Estonia;

CPCM – Collection of Peter Chandler, Melksham, U.K.

## Taxon treatments

### Ditomyia macroptera

(Winnertz, 1852)

38A8A92A-96E9-5655-A89B-3E361082D42E

#### Materials

- **Type status:** Other material. **Occurrence:** recordedBy: Urmas Jürivete; individualCount: 1 male 2 females; **Taxon:** scientificName: *Ditomyiamacroptera* (Winnertz, 1852); **Location:** country: Bulgaria; countryCode: Bulgaria/BG; stateProvince: Blagoevgrad; municipality: Strumyani; locality: Ilindentsi, old orchard; verbatimCoordinates: 41°39"N, 23°14"E; coordinatePrecision: 0.01667; **Identification:** identifiedBy: Olavi Kurina; **Event:** samplingProtocol: reared; eventDate: 2014-04-25/2014-05-05; eventRemarks: reared from *Phellinusalni*, coll 25.04.2014, emerged 05.05.2014; **Record Level:** collectionCode: IZBE; basisOfRecord: PreservedSpecimen
- **Type status:** Other material. **Occurrence:** recordedBy: Phil Withers; individualCount: 1 male; **Taxon:** scientificName: *Ditomyiamacroptera* (Winnertz, 1852); **Location:** country: France; countryCode: France/FR; stateProvince: Bourgogne-Franche-Comté; municipality: Doubs; locality: Lac de Remoray, bas marais du Crossat; **Identification:** identifiedBy: Peter Chandler; **Event:** samplingProtocol: Malaise trap; eventDate: 2011-04-26; eventRemarks: ex Malaise trap No 9; **Record Level:** collectionCode: CPCM; basisOfRecord: PreservedSpecimen
- **Type status:** Other material. **Occurrence:** recordedBy: Phil Withers; individualCount: 1 female; **Taxon:** scientificName: *Ditomyiamacroptera* (Winnertz, 1852); **Location:** country: France; countryCode: France/FR; stateProvince: Bourgogne-Franche-Comté; municipality: Doubs; locality: Lac de Remoray, bas marais du Crossat; **Identification:** identifiedBy: Peter Chandler; **Event:** samplingProtocol: Malaise trap; eventDate: 2011-08-09; eventRemarks: ex Malaise trap No 10; **Record Level:** collectionCode: CPCM; basisOfRecord: PreservedSpecimen

#### Diagnosis

**Figure 2.**
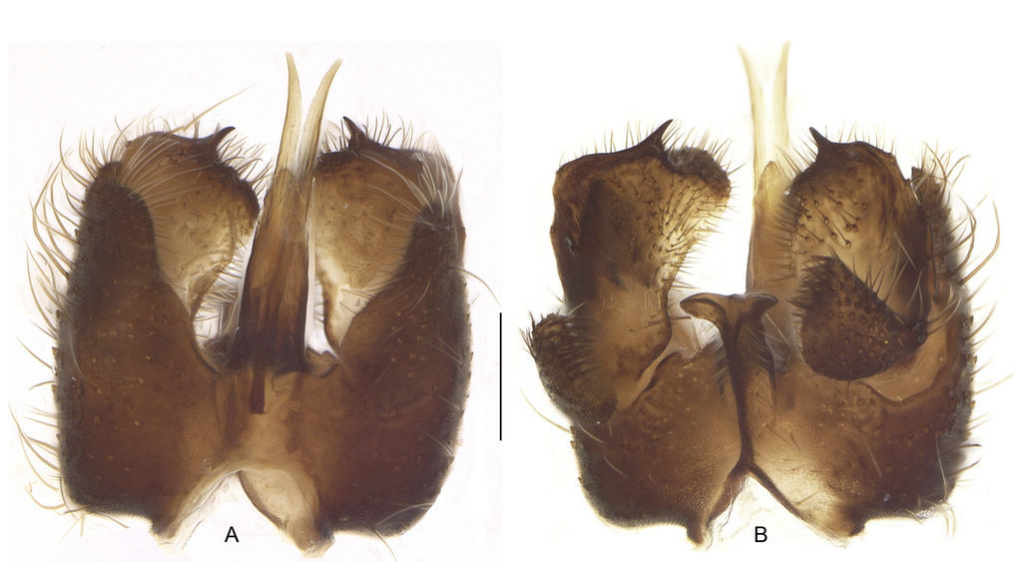
*Ditomyiamacroptera* Winnertz, male terminalia: dorsal view (A), ventral view (B). Scale bar 0.2 mm.

The imago of *D.macroptera* (Fig. 1) is large (up to 8 mm; about 5 mm in *D.fasciata*), dark brown to blackish (yellowish-brown in *D.fasciata*), while the wings are uniformly smoky (smoky with 2 light transverse bands in *D.fasciata*, cf. Kurina and Grootaert 2016: fig. 5A). The male and female terminalia are previously figured by Zaitzev (Zaitzev 1978: fig. 6, Zaitzev 1994: fig. 24-3,7). The male terminalia of the studied specimen from Bulgaria are provided in lateral (Fig. 1), dorsal and ventral views (Fig. 2).

#### Conservation

Due to its rarity, *D.macroptera* is considered as critically endangered (CR) in the Czech Republic (Ševčík 2005).

#### Biology

According to Landrock (Landrock 1940) the species is rare in the mountain forests of Central Europe. This species has only been reared from two very similar species of bracket fungi: *Phellinusigniarius* (Winnertz 1852) and *Ph.alni* (original data).

## Discussion

**Figure 3.**
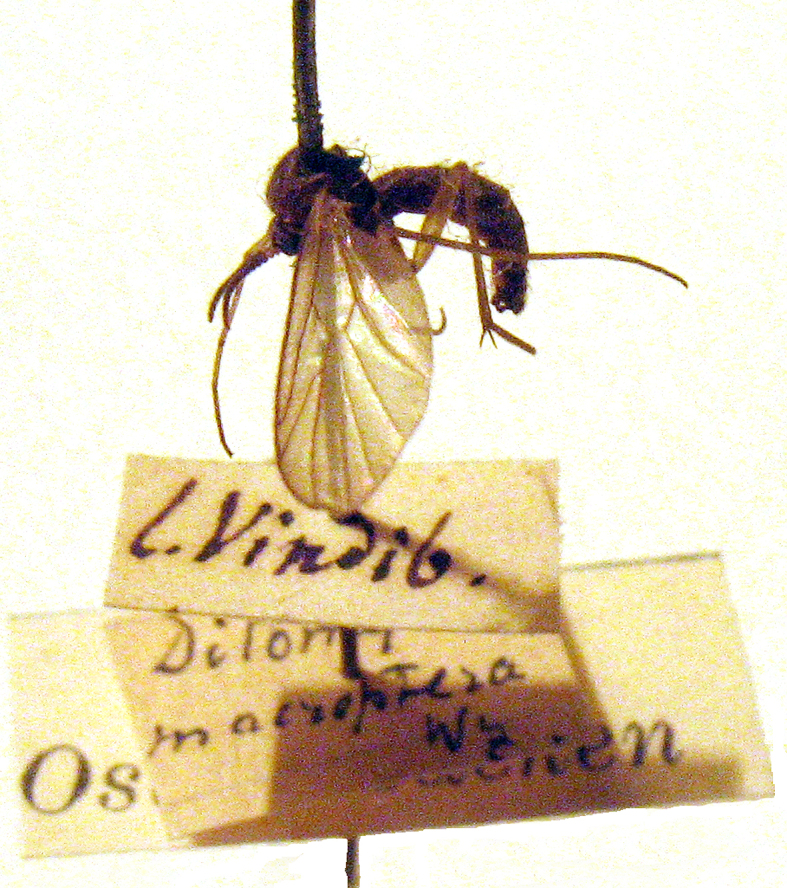
*Ditomyiamacroptera* Winnertz, male specimen in the collection of C.R. Osten-Sacken in Zoological Institute, Russian Academy of Sciences, St. Petersburg. Photo by A. Przhiboro.

The type of *D.macroptera* is probably destroyed, like most of the material collected by J. Winnertz, when it was stored in Poppelsdorf Castle near Bonn (Germany) during World War II (Evenhuis 1997). Some of Winnertz’ Diptera types have, however, survived in the Senckenberg Museum of Natural History, Frankfurt a. M., Germany (cf. Plassmann 1970) and in the Natural History Museum Vienna, Austria (cf. http://www.nhm-wien.ac.at/en/museum) but *D.macroptera* is not listed. Zaitzev (Zaitzev 1994: 35) characterised the species according to one male and one female specimen from the collection of C.R. Osten-Sacken in ZIN (Zoological Institute, Russian Academy of Sciences, St. Petersburg), with a note that the collecting locality for them was unknown but with a collecting date as early as 11.v.1835 (i.e. 17 years before the description of the species). At present, the collection of Osten-Sacken housed in ZIN includes one male specimen (Fig. 3) with a label “L. Vindib.” but without collecting date included (A. Przhiboro *pers. comm.*). We are not aware of the location of the female specimen that is (was) apparently supplied also with the date label mentioned by Zaitzev (Zaitzev 1994: 35). The specimens of *D.macroptera* in the collection of Osten-Sacken were probably acquired from the remains of the P.C. Zeller collection after 1876, when C.R. Osten-Sacken returned to Europe (cf. Osten-Sacken 1903: 9–10). However, the label is different from most of those under specimens collected by Zeller (A. Pont *pers. comm.*). Otherwise, Horn et al. (Horn et al. 1990: Plate 5, fig. 27) provided a handwritten label by Zeller with “Mus. Vindob.” on it, representing the Vienna Museum. At the present stage of knowledge, we are not able to clarify the mystery of two specimens in the Osten-Sacken collection, but with a high probability, they have been collected in Central Europe.

The Belgian record (Gosseries 1991: 65) is based on a female specimen (from Munte near Gent, 11.vii.1929, M. Goetghebuer leg.) in the collection of RBINS [Royal Belgian Institute of Natural Sciences, Brussels] that proved to be, after thorough recent studies, *Symmerusannulatus* (Meigen, 1830). Consequently, the distribution range of *D.macroptera* should be restricted to exclude Belgium.

We were not able to study the single specimen from the Eastern Palaearctic (Okada 1936) and the record remains questionable. At the time when Okada studied the specimen, there were neither detailed description nor figures of male or female terminalia of *D.macroptera* available. Subsequently, five additional *Ditomyia* species were described from the Eastern Palaearctic. Three of them, viz. *D.claripennis* Saigusa, 1973, *D.spinifera* Zaitzev, 1978 and *D.insularis* Zaitzev, 1994, have been discussed as resembling *D.macroptera* (Saigusa 1973, Zaitzev 1978, Zaitzev 1994). The record of *D.macropt*era from Sakhalin Island was questioned also by Zaitzev (Zaitzev 1994).

Our records are from European mountain areas that corroborate earlier characterisation on habitat requirements of the species (e.g. Landrock 1940). The Bulgarian record, in addition to being the southernmost so far, also represents the second record of its larval host after one and half centuries.

## Supplementary Material

XML Treatment for Ditomyia macroptera
